# Microencapsulation of Phenolic Extracts from Cocoa Shells to Enrich Chocolate Bars

**DOI:** 10.1007/s11130-021-00917-4

**Published:** 2021-09-06

**Authors:** M. Grassia, M.C. Messia, E. Marconi, Ȫ. Şakiyan Demirkol, F. Erdoğdu, F. Sarghini, L. Cinquanta, O. Corona, D. Planeta

**Affiliations:** 1 Department of Agricultural, Environmental and Food Sciences, University of Molise, Via F. De Sanctis, 86100 Campobasso, Italy; 2 Department of Food Engineering, Ankara University, 06830 Ankara, Turkey; 3 Department of Agricultural Sciences, Università Degli Studi di Napoli Federico II, Via Università, 100, 80055 Portici, NA Italy; 4 Department of Agricultural, Food and Forest Sciences, University of Palermo, Viale delle Scienze 4, 90128 Palermo, Italy

**Keywords:** Cocoa, Encapsulation, Polyphenols, Shelf life, Spray drying

## Abstract

Cocoa bean shells were subjected to green extraction technologies, based on the absence of toxic organic solvents, to recover polyphenols; the extract was then encapsulated using a spray dryer and maltodextrin as coating agent. The best conditions observed in the spray drying tests (core-to-coating ratio 1:5; inlet temperature 150 °C; flow rate 6 ml min^−1^) were applied to produce the microcapsules used to enrich the same cocoa mass as the shells and processed for the preparation of the chocolate bars. Sensory analysis showed no significant differences between enriched chocolate bar and the unenriched reference one, except for the appearance. Both samples were then subjected to accelerated storage tests, at the end of which the polyphenols in the control chocolate bar (0.85 g 100 g^−1^) were reduced by about 50% (0.42 g 100 g^−1^), while in the enriched chocolate (1.17 g 100 g^−1^) by only 22% (0.97 g 100 g^−1^). The proposed process significantly enriched the chocolate bars with phenolic antioxidants recovered from cocoa waste without increasing the sensations of bitterness and astringency.

## Introduction

Today the recovery and reuse of by-products in the perspective of circular economy is necessary in view of the increasing world population and disposal costs [1, 2]. Cocoa shells make up 12% of the whole bean and are usually considered a waste product of cocoa manufacturing. From the annual production of cocoa beans (4,824,000 tons in 2019/2020), approximately 578,900 tons of food shells are produced (https://www.statista.com/statistics/262620/global-cocoa-production/). Cocoa shells are a source of antioxidant compounds, such as polyphenols, including epicatechin, catechin and procyanidins [3–6], flavanols, in particular, have been shown to protect against oxidative stress in cell cultures and animal models [7]; with color and flavor similar to chocolate powder [8] and rich in dietary fiber. Usually, their traditional extraction techniques require high amounts of solvents and heating [8], otherwise, green extraction is based on the discovery and design of extraction processes that allow use of alternative solvents and renewable natural products, as well as ensure a safe and high quality of extract/product. In particular, it is important to avoid heating and the use of solvents harmful to human health and the environment. Several techniques can be used to protect the phenolic extract, including: extrusion coating, fluidized bed coating, inclusion complexation, freeze drying and spray drying. The process of microencapsulation, which involves covering it with a coating material [9], is an effective process for exploiting the polyphenolic extract [10, 11]. Currently, due to increasing health concerns, the functional food market is experiencing exponential growth. As a result, many traditional products have been used as a matrix to introduce bioactive compounds into the diet, providing both nutritional and functional benefits from food. For the first time, to the best of our knowledge, a process of encapsulation of phenolic extracts from cocoa shells to enrich the chocolate bar obtained from the same cocoa, has been developed. The purpose of the research was to increase the antioxidant properties of chocolate, masking the bitter and astringent taste. Therefore, the green extraction process of phenolic substances from cocoa shells was first investigated and the extracts were encapsulated with maltodextrins by spray drying. Microcapsules with phenolic extracts from cocoa shells were then added during the hardening of cocoa liquor for the production of chocolate bars. The resulting products were evaluated for their antioxidant capacity and sensory properties. The enriched chocolate bars were finally subjected to an accelerated shelf life test and analysed for phenol content, colour and texture.

Key words: cocoa shell; micro-encapsulation; spray drying; phenols; enriched chocolate; shelf life

## Materials and Methods

### Proximate Analysis of Cocoa Beans and Shells

Cocoa beans from the Ivory Coast (var. Forastero), and shells, by shelling machine during cocoa manufacturing process, were collected from the company Dolceamaro srl (Monteroduni, Italy). The samples were analysed for moisture, ash, protein, total fat, fiber and total phenols. Available carbohydrate content was estimated by difference. Moisture content of the samples was determined by employing the gravimetric method, drying 5 g of the ground sample at 101 ± 2 °C to constant weight in an air oven. Water activity (Aw) was measured using an AquaLab CX3-TE apparatus (Labo-Scientifica, Parma, Italy). Ash content was determined by using a muffle furnace at 550–600 °C up to white ash. Fat content of the chocolate samples was carried out by the Soxhlet extraction method according to AOAC [12]. The protein content was determined based on total nitrogen content measured with the Kjeldahl procedure, multiplied by a factor of 6.25 (protein = nitrogen x 6.25). The chemical composition of total, soluble and insoluble dietary cocoa fiber was established according to AOAC [13].

### Phenolic Extraction from Cocoa Shells

Polyphenols were extracted from cocoa shells following different protocols. Water, ethanol, methanol (80%), and water-ethanol solutions (75, 50, and 25%) were used. A 2210 Bransonic ultrasonic bath (125 kW- 47 kHz ± 6%) was used to facilitate the extraction of phenols. The extractions were performed as described elsewhere [14]. Six groups of ground shells with different particle size (*μ*m) were studied to enhance phenol extraction, the sizes of shells were separated using sieves with different size range: (φ) >1,000; >850; >710; >500; >250; <250 *μ*m. Once the best extraction conditions were determined (based on solvent and extraction time on ground cocoa shells with a particle size of 250 *μ*m), shells were passed through six different sieves. All samples were extracted in triplicate.

### Total Phenolic Content (TPC)

TPC were determined by the Folin Ciocolteu method, using catechin as a standard. Phenolic compounds were extracted three times with methanol/water (4:1, v/v) at 50 °C for 2 h. Then, samples were centrifuged (5 min, 5,000 *g*) decanted and supernatants were pooled [15].

All samples were prepared in triplicate.

### Encapsulation of Phenolic Extract by Spray Drying

The phenolic extract was encapsulated using maltodextrin, dextrose equivalent (DE):17.0–19.9, purchased from A.C.E.F. (Fiorenzuola D’Arda - PC, Italy), as a coating materials. The solutions (10%, w/v) in water were prepared 1 day before the encapsulation process [16]. For encapsulation, 1 g of lyophilized polyphenols (Genesis 25SES, VirTis Co., Gardiner, NY), were mixed with maltodextrin at three different concentrations (core-to-coating ratio 1:5; 1:10; and 1:15 W:W), the mixtures were then homogenized at 7,000 rpm for 10 min using a high-speed homogenizer (DH. WHG02118, Daihan Scientific CO., Ltd. Korea). The micro-encapsulation was carried out using a Mini Spray Dryer B-290 (Buchi Labortechnic AG, Switzerland) equipped with a co-current two-fluid nozzle. The different mixtures were fed at a speed of 6 and 9 mL min^−1^; the drying was performed at two different inlet temperatures: 120 and 150 °C with an aspiration rate of 28 m^3^ h^−1^. In brief, twelve samples subjected to spray drying were compared (3 different core:coating ratios × 2 feed rates × 2 inlet temperatures).

### Encapsulated Phenolic Extract Yield

The dried yield was calculated using Eq. (1)

1 $$ Yield\ \left(\%\right)=\mathrm{Final}\ \mathrm{mass}\ \mathrm{of}\ \mathrm{dried}\ \mathrm{microcapsules}\ \left[\mathrm{g}\right]/\mathrm{Initial}\kern0.5em \mathrm{mass}\ \mathrm{of}\ \mathrm{feed}\ \mathrm{solution}\ \left[\mathrm{g}\right]\times 100 $$

### Surface Phenolic Content (SPC)

100 mg of microcapsules were treated with 1 mL of a mixture of ethanol and methanol (1:1). These solutions were agitated in a Vortex at room temperature for 1 min and then filtered (0.45 *μ*m Millipore filter) [11]. The amount of phenolic compounds were quantified by the Folin–Ciocalteu method.

### Antioxidant Activity (AA)

The antioxidant activity (AA) was determined using the DPPH method: in its radical form, DPPH absorbs at 515 nm, but upon reduction by an antioxidant or a radical species, its absorption disappears. The samples were analysed as reported elsewhere [17] and the AA was expressed as percentage inhibition of DPPH.

### Particle Size Analysis

Particle size distribution of the capsules was measured by a Malvern Mastersizer 3000E Hydro, (Malvern Instruments, Worcestershire, UK). Each particle size measurement was made from the average of three readings *per* sample. The Mastersizer was used by a laser obscuration greater than 10%; tests were repeated three times for each run. Particle size was expressed as the average volume-weighted diameter D [3, 4].

### Colour Properties

Colour parameters (L*, a*, b*) were measured with CR-400 Chroma Meter (Minolta, Osaka) equipment. The Hue angle H (°) and the Chroma C* values were calculated as follow:

2 $$ \mathrm{H}\left({}^{\circ}\right)={\tan}^{-1}\left({\mathrm{b}}^{\ast }/{\mathrm{a}}^{\ast}\right) $$

3 $$ \mathrm{C}\ast =\surd {\left(a\ast \right)}^2+{\left(b\ast \right)}^2 $$

where a*, b*: chromaticity coordinates in the L*a*b* color space.

### Production of Chocolate Enriched with Shell Polyphenols

The microcapsules obtained by spray drying with the best characteristics (indicated below as S3) were added during the production of chocolate bars, after the tempering step, at Dolceamaro firm (Monteroduni, Italy) [18]. The goal was to exceed 1% of total polyphenols in chocolate bars.

### Sensorial Analysis

The evaluation of the sensory profiles of chocolate bars was performed on freshly made chocolate bars (enriched and not), following the ISO sensory analysis [19]. Twelve panelists (three males and nine females, aged 25–50 years) were specifically trained by expert chocolate tasters. The sensory attributes cited with a frequency higher than 60% by panelists were selected for sample evaluation. Thirteen descriptors were included in the analysis. The two samples were randomly evaluated assigning a variable scoring scale *per* descriptor, with the lowest values indicating no sensation and the highest values extremely intense [20].

### Accelerated Shelf Life Assessment

An accelerated storage test was then conducted to predict the shelf life of the chocolate bars enriched with polyphenols (P) and the control samples (C), packed with PP film, were stored in an incubator at isothermal conditions of 25 °C [21], at RH = 70% for 56 days. One week of accelerated storage corresponds to one month of shelf life [22].

### Texture Analysis

The sample texture was analyzed with a TA-XT2 texture analyzer (Texture Technologies Corp., Scardsale, NY) and Texture Expert Software. A 6 mm cylinder probe was used to simulate the compression of a bite. The conditions for the test were: pretest speed of 2 mm s^−1^, test speed of 2 mm s^−1^, post-test speed of 5 mm s^−1^, distance of 90%, relaxation time of 5 s and force of 50 g. Dimensions of chocolate pieces were 20 mm X 20 mm X 20 mm (l x w x h). The parameters evaluated were hardness and adhesiveness. The results represent the average of three analyses.

### Statistical Analysis

Analysis of variance (ANOVA) and Tukey’s multiple range test for *p* ≤ 0.05 were used to investigate the differences between all the data. All statistical analyses were done via the SPSS Version 20.0 statistic software package (SPSS Inc., Chicago, IL, USA).

## Results and Discussion

### Extraction of Phenols from Cocoa Beans

**Fig. 1:**
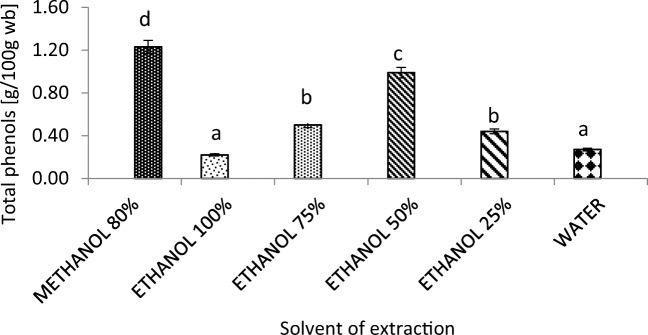
Effect of different solvents on extraction yields of total polyphenols from cocoa shells (particle size = 250 *μ*m). Different letter indicate significant difference (*P* < 0.05). Values are mean ± standard deviation of triplicates

**Fig. 2:**
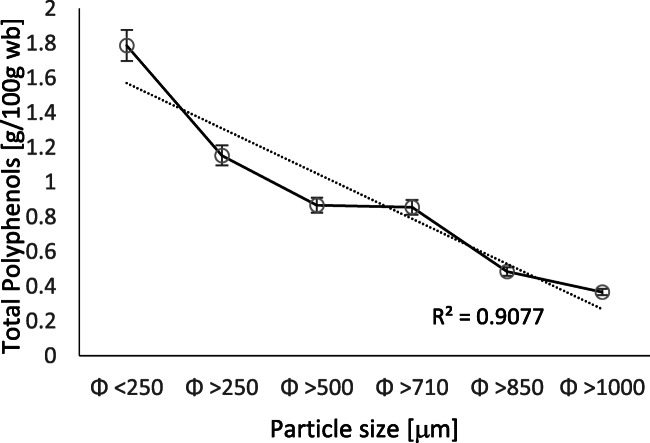
Effect of cocoa shell particle size on polyphenol extraction yield using 50% ethanol in water. Values are mean ± standard deviation of triplicates. The dotted line represents the interpolation between the experimental points

The chemical composition of cocoa beans and their shells on dry basis (d.b.) showed (Table 1) the latter to have slightly more than half the polyphenol content of cocoa beans (1.2 g 100 g^−1^ d.b.) and almost three times the fiber content (48.5 g 100 g^−1^ d.b.). Extraction of polyphenols from cocoa shells showed higher yields with the solvent composed of 50% ethanol in water (Fig. 1), not considering the 80% methanol solvent because of its toxicity. When evaluating the influence of particle size of ground cocoa shells on extraction yield, it was found that the larger the particle size, the lower the amount of total phenols extracted. The results showed an inverse correlation (R^2^ = 0.91) between the two variables described above (Fig. 2). Extraction with smaller particle sizes caused higher yields, as the smaller particle sizes provided more surface area for mass transfer. At the end of the extraction tests, the best conditions for the green recovery of phenolic compounds from the cocoa shell were as follows: 50% ethanol solution in water for 15 min, with raw material particle size <250 *μ*m, under ultrasonically assisted condition. Other authors have optimized the extraction process using the following parameters: 100 °C, 90 min, water, 0.02 g cocoa shell mL^−1^ [23], and evaluated cocoa shell and its bio-compounds [24].

**Table 1:** Chemical composition (g 100 g^−**1**^) of cocoa beans and cocoa shells on dry basis

Sample	Moisture	Total Fat	Available Carbo-hydrates	Proteins	Dietary Fibers	Ash	Total Phenols
Cocoa beans	7.1 ± 0.8	58.1 ± 0.5	4.1	14.0 ± 0.3	17.7 ± 0.5	3.6 ± 0.2	2.5 ± 0.2
Cocoa shells	3.3 ± 0.1	14.0 ± 0.61	16.1	14.4 ± 0.2	48.5 ± 0.4	5.8 ± 0.3	1.2 ± 0.1

Values are mean ± standard deviation of triplicates

### Spray-Drying and Encapsulation

**Fig. 3:**
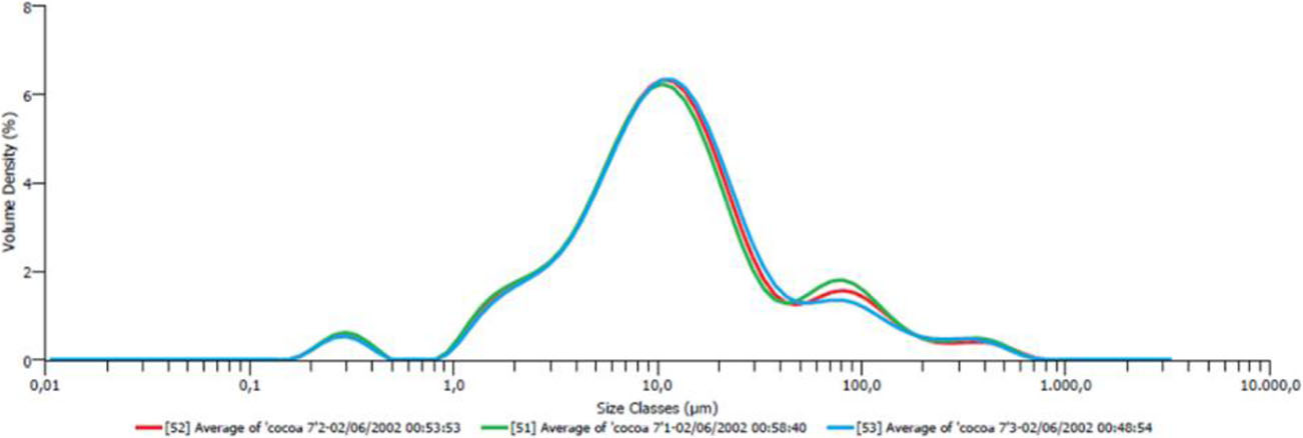
**-** Size distribution of powder encapsulated with maltodextrin by spray drying in sample S3 (see Table 3 for coding). The curves represent three repetitions

The 12 different encapsulation trials during spray drying are shown and coded in Table 2. The yield (Y) of dried powder formed from phenolic extract encapsulated with maltodextrin, after spray drying was highest at 150 °C, with a flow rate of 6 mL min^−1^ (Y = 81% in sample S11 and 78% in S3) (Table 2). Moreover, increasing the feed rate decreased the outlet temperatures (Table 2), while no correlation between yield and the core-to-coating ratio was found. Moisture content was inversely proportional to drying temperature (Table 3), the increase in which caused a higher rate of water evaporation. The higher moisture in microcapsules was related to the flow rate (Table 3). The higher the flow rate, the less time the feed liquid is in contact with the hot air and therefore eventually results in higher moisture. The total polyphenol content (TPC) was the highest at a 1:5 core: maltodextrin ratio [25], with no significant variation due to inlet temperature and feed rate (Table 3). Surface polyphenol content was lowest in sample S3 (about 5% TPC), followed by S2 (about 8% TPC) (data not reported). Antioxidant activity (AA) values varied significantly among the three groups with different concentrations in the core-to-coating ratio (AA = 27.0% for ratio of 1:15; AA = 52.3% for ratio of 1:10 and AA = 63.7% for ratio of 1:5). In summary, considering the total polyphenol content, encapsulation yield and antioxidant activity, the best results in spray drying were obtained with the 1:5 core: maltodextrin ratio. Among these samples, S3 showed the highest yield and lowest moisture content. The influence of different spray drying operating conditions was also evaluated by measuring particle size D[4:3] (mean volume momentum), D10 (10th percentile size), D50 (median size), and D90 (90th percentile size) values (Table 4). The encapsulated powder produced from maltodextrin showed a trimodal distribution with three sizes: approximately at 0.5 *μ*m, 10 *μ*m and 80 *μ*m (Fig. 3). The microcapsules obtained in sample S10 (D[4:3] = 19.5) and S3 (D[4:3] = 30.6) were characterized by a satisfactory particle size distribution, considering that a good dark chocolate has its own maximum particle size of 30 *μ*m, otherwise it looks grainy [26].

**Table 2:** Dried yield of microcapsules obtained by spray drying at different core: maltodextrin ratios, inlet and outlet temperatures, and flow rate.

Sample coding	Ratio core: coating agent	Inlet temperature [°C]	Outlet temperature [°C]	Flow rate [ml/min]	Yield *[%]
S1	1:5	120	67	6	71.9
S2	1:5	120	51	9	74.0
S3	1:5	150	87	6	78.1
S4	1:5	150	71	9	59.4
S5	1:10	120	62	6	73.3
S6	1:10	120	50	9	61.2
S7	1:10	150	87	6	68.7
S8	1:10	150	72	9	68.8
S9	1:15	120	62	6	78.0
S10	1:15	120	57	9	70.9
S11	1:15	150	73	6	80.7
S12	1:15	150	60	9	76.3

*Values are mean ± standard deviation of triplicates

**Table 3:** Moisture, total polyphenols (TPC), and antioxidant activity (DPPH) in phenolic extract samples microencapsulated by different operating parameters

Sample	Moisture (%)	TPC (mg/kg)	AA %
S1	4.0 ± 0.3^c^	21.9 ± 0.9 ^c^	64.4 ± 1.9^d^
S2	5.3 ± 0.3^d^	23.0 ± 1.3 ^c^	63.2 ± 2.3^d^
S3	3.9 ± 0.3^c^	22.7 ± 1.1^c^	64.0 ± 2.1^d^
S4	5.7 ± 0.4^d^	26.2 ± 0.6^c^	63.4 ± 2.6^d^
S5	3.8 ± 0.1^c^	8.8 ± 0.8^a^	52.0 ± 1.9^c^
S6	4.3 ± 0.2^c^	8.6 ± 0.4^a^	52.7 ± 1.4^c^
S7	3.6 ± 0.1^c^	11.0 ± 0.2^b^	52.7 ± 1.2^c^
S8	4.4 ± 0.2^c^	12.8 ± 0.4^b^	51.9 ± 2.2^c^
S9	3.2 ± 0.2^b^	8.6 ± 0.9^a^	28.5 ± 1.9^b^
S10	3.9 ± 0.1^c^	7.1 ± 0.4^a^	27.4 ± 1.4^b^
S11	2.4 ± 0.1^a^	7.4 ± 0.9^a^	31.0 ± 1.9^b^
S12	3.1 ± 0.2^b^	7.5 ± 0.4^a^	21.0 ± 1.4^a^

Values are mean ± standard deviation of triplicates

Different letters on the same column represent significant differences (*P* < 0.05)

**Table 4:** Particle size (*μ*m) and volume distribution D [3, 4] of the phenolic extract samples microencapsulated by different operating parameters

Sample	Dx(10)	Dx(50)	Dx(90)	D [3, 4]
S1	3.4 ± 0.2	41 ± 1.1	171 ± 2.3	68.5 ± 1.2
S2	1.7 ± 0.1	14.1 ± 0.5	115 ± 1.9	42.6 ± 0.7
S3	2.4 ± 0.1	10.9 ± 0.4	74.6 ± 1.0	30.6 ± 1.1
S4	3.2 ± 0.2	12.1 ± 0.3	87 ± 2.2	40.3 ± 0.5
S5	2.0 ± 0.1	21.3 ± 0.4	117 ± 1.1	44.4 ± 0.7
S6	2.5 ± 0.1	25.2 ± 0.3	102 ± 1.3	38.9 ± 0.5
S7	2.2 ± 0.1	28.1 ± 0.6	122 ± 1.1	42.6 ± 0.3
S8	2.8 ± 0.1	24.6 ± 0.5	112 ± 2.3	45.7 ± 0.6
S9	2.5 ± 0.1	16.9 ± 0.3	133 ± 1.5	48.5 ± 0.8
S10	2.5 ± 0.1	11.6 ± 0.4	49.6 ± 0.3	19.5 ± 0.3
S11	2.3 ± 0.1	20 ± 0.7	209 ± 0.5	95.1 ± 0.6
S12	2.7 ± 0.1	16.8 ± 0.6	77 ± 0.2	52.1 ± 0.5

Values are mean ± standard deviation of triplicates

### Sensorial Analysis

**Fig. 4:**
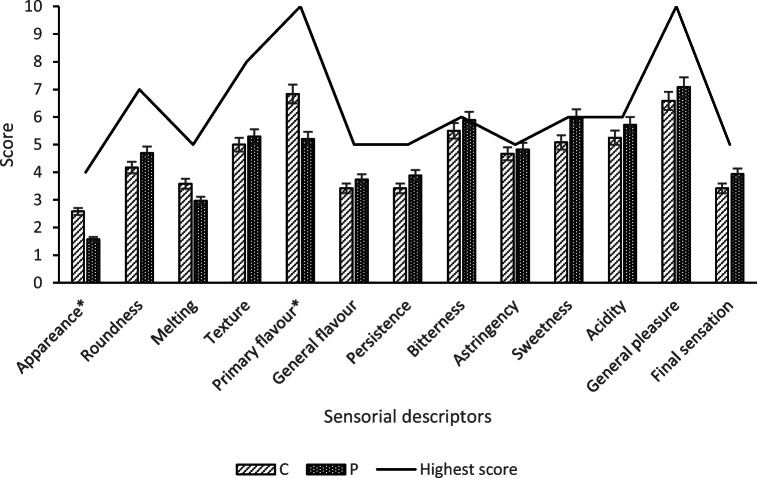
**-** Sensory profile of the control (C) and enriched (P) chocolate bars. Final score: (C) =70/100; (P) = 73/100. For each descriptor * indicate significantly different at *P* < 0.05

On the 13 sensory descriptors, the only significant differences between the enriched samples and the control were related to appearance and primary aromas. The appearance of the samples (P) showed more irregularity, probably due to the different sizes and shapes of the microcapsules. The primary aroma was rated better in (C) than in (P) samples, perhaps because the maltodextrin had partially masked the cocoa flavour (Fig. 4). It is of interest that no significant differences were found in sweetness, bitterness, astringency, and acidity, despite the increase in polyphenols in the enriched samples. Finally, based on the results of the study, both samples (C and P) can be considered as medium-high quality chocolate bars, with a final score of 70/100 and 73/100, respectively (Fig. 4).

### Chocolate Bars and Accelerated Shelf Life

An accelerated storage test was conducted to predict the shelf life of polyphenol-enriched chocolate bars added with microcapsule S3 (P) and the control samples (C), consisting of the same unenriched chocolate. Every two weeks throughout an 8-week period, on both samples the following analyses were performed: moisture content, water activity, total phenols, color, texture and sensory analysis. Moisture content in both samples was within the worth limits of chocolate [27], ranging from 0.8 to 1.5%. In both chocolate bars up to 42 days of accelerated shelf life, water activity (Aw) values were < 0.3. During the accelerated shelf life test, the samples (C) and (P) did not show different L* and Chroma values, while Hue angle varied at 0 and 28 days (Table 5). During the first 28 days of storage, (P) showed lower values of a* and b*, compared with the control, thereafter no significant differences were shown. The enriched chocolate had significantly higher phenol content than the control: at time 0, the total amount of phenols in (P) was 1.2 g/100 g instead of (C) that contained 0.85 g/100 g. At the end of the accelerated shelf life trials, phenols in control chocolate (C) were reduced to about 50% while in (P) only to 22%. This difference is a consequence of the encapsulation technique that protected phenolic compounds from oxidative degradation. Several studies [28, 29] have already observed the depletion of polyphenols in cocoa-based products during storage. In another research [30], a decrease in total phenols of 64% after eighteen months of storage under conditions simulating a point of sale was found.

**Table 5:** Color parameters during chocolate bar accelerated shelf life test. (S) = Control - (P) = Enriched chocolate

days	Sample	L*	a*	b*	Hue angle (°)	Chroma
0	C	29.6 ± 0.2^a^	6.2 ± 0.1^b^	4.6 ± 0.1^b^	1.1 ± 0.1^a^	7.7 ± 0.1^a^
	P	28.6 ± 0.1^a^	5.7 ± 0.2^a^	3.5 ± 0.1^a^	1.4 ± 0.1^b^	6.7 ± 0.1^a^
14	C	29.7 ± 0.1^a^	6.2 ± 0.1^b^	4.9 ± 0.1^b^	1.0 ± 0.1^a^	7.9 ± 0.1^a^
	P	28.9 ± 0.4^a^	5.5 ± 0.1^a^	3.4 ± 0.4^a^	1.4 ± 0.1^a^	6.4 ± 0.2^a^
28	C	29.6 ± 1.4^a^	6.2 ± 0.2^b^	4.4 ± 0.6^a^	1.2 ± 0.2^a^	7.6 ± 0.3^a^
	P	31.9 ± 0.1^a^	5.5 ± 0.1 ^a^	5.4 ± 0.2^a^	0.7 ± 0.1^b^	7.7 ± 0.2^a^
42	C	29.7 ± 0.6^a^	5.9 ± 0.2^a^	4.1 ± 0.3^a^	1.2 ± 0.1^a^	7.2 ± 0.3^a^
	P	30.3 ± 0.7^a^	6.3 ± 0.5^a^	4.9 ± 0.3^b^	1.0 ± 0.1^a^	7.9 ± 0.5^a^
56	C	29.2 ± 0.1^a^	6.3 ± 0.1^a^	4.6 ± 0.1^a^	1.1 ± 0.1^a^	7.8 ± 0.1^a^
	P	29.2 ± 0.7^a^	6.4 ± 0.4^a^	4.5 ± 0.3^a^	1.2 ± 0.1^a^	7.8 ± 0.5^a^

Values are mean ± standard deviation of triplicates

Different letters on the same column for pairs of values represent significant differences (*P* < 0.05)

### Texture Analysis

Hardness is correlated with the crystalline microstructure and particle size distribution in the chocolate bars: smaller particle size leads to an increase in hardness [31], meantime chocolate bars with a particle size <20* μ*m are perceived by consumers to be smooth in texture [32]. In the (P) samples hardness decreased significantly in the first 42 days, while the (C) samples showed a smaller reduction (Table 6). In general, storing dark chocolate at high temperatures causes a decrease in hardness [33]. At the beginning of the observation, the adhesiveness was the same in both samples, while it was lower during the entire storage in the control samples. In (C) the adhesiveness decreased more than in (P) over the course of storage (Table 6), probably due to less moisture absorption. It is noteworthy that the decrease in the negative values of adhesiveness indicates an increase in its intensity.

**Table 6:** Water activity (Aw); Total phenols (mg/kg); Hardness (g) and Adhesiveness (g) during chocolate bar accelerated shelf life. (C) = Control; (P) = Enriched chocolate

days	Aw (C)	Aw (P)	Phenols (C)	Phenols (P)	Hardness (C)	Hardness (P)	Adhesiveness (C)	Adhesiveness (P)
0	0.27 ± 0.01^a^	0.23 ± 0.01^a^	854 ± 29^a^	1169 ± 60^b^	11,269 ± 1481^a^	30,798 ± 2059^b^	−0.27 ± 0.17^a^	−0.26 ± 0.04^a^
14	0.29 ± 0.13^a^	0.26 ± 0.01^a^	780 ± 12^a^	1076 ± 18^b^	11,033 ± 319^a^	24,211 ± 1677^b^	−1.97 ± 0.12^a^	−0.35 ± 0.06^b^
28	0.29 ± 0.01^a^	0.28 ± 0.01^a^	683 ± 21^a^	1069 ± 77^b^	9976 ± 2022^a^	16,170 ± 2004^b^	−2.46 ± 0.06^a^	−0.44 ± 0.18^b^
42	0.23 ± 0.01^a^	0.22 ± 0.01^a^	634 ± 12^a^	992 ± 15^b^	8607 ± 1010^a^	12,842 ± 113^b^	−3.70 ± 0.18^a^	−0.59 ± 0.01^b^
56	0.35 ± 0.01^b^	0.31 ± 0.01^a^	424 ± 15^a^	970 ± 30^b^	8442 ± 106^a^	11,088 ± 149^b^	−4.06 ± 0.03^a^	−0.54 ± 0.02^b^

Values are mean ± standard deviation of triplicates

Different letters on the same row for pairs of values represent significant differences (*P* < 0.05)

## Conclusions

Following green extraction to recover polyphenols from cocoa bean shells, the extracts were encapsulated by spray drying with maltodextrin as a coating agent. The best conditions observed in the spray-drying tests were applied to produce the microcapsules, which were used to enrich the same cocoa mass as the shells, and processed for the preparation of chocolate bars. The enriched chocolate bar showed a significant increase in phenols compared to the control one, and the presence of maltodextrin prevented the perception of bitterness and astringency. At the end of the accelerated storage test, both chocolate bars obtained a satisfactory score in the sensorial test. The only defects found in the sensorial analysis at the end of the accelerated shelf life of enriched chocolate were regarding appearance and primary aromas. The proposed encapsulation process allowed to enrich chocolate bars with antioxidant substances extracted from shells, without increasing negative taste perceptions.

The datasets generated during and/or analysed during the current study are available from the corresponding author on reasonable request.

### Data Availability Statements

The datasets generated during and/or analysed during the current study are available from the corresponding author on reasonable request.
